# Cardiovascular Health and Biomarkers of Neurodegenerative Disease in Older Adults

**DOI:** 10.1001/jamanetworkopen.2025.0527

**Published:** 2025-03-11

**Authors:** Anisa Dhana, Charles S. DeCarli, Klodian Dhana, Pankaja Desai, Ted K. S. Ng, Denis A. Evans, Kumar B. Rajan

**Affiliations:** 1 Rush Institute for Healthy Aging, Rush University Medical Center, Chicago, Illinois; 2 Department of Internal Medicine, Rush University Medical Center, Chicago, Illinois; 3 Department of Neurology, University of California, Davis, Sacramento

## Abstract

**Importance:**

Cardiovascular health (CVH), defined by the American Heart Association as Life’s Simple 7 to promote a healthy lifestyle and manage vascular risk factors, has been associated with a low risk of Alzheimer disease and less vascular dementia. However, the association between CVH and biomarkers of neurodegeneration remains less understood.

**Objective:**

To investigate the association of CVH with serum biomarkers of neurodegeneration, including neurofilament light chain (NfL) and total tau (t-tau).

**Design, Setting, and Participants:**

This cohort study was conducted within the biracial, population-based Chicago Health and Aging Project (CHAP) of adults aged 65 years or older between 1993 and 2012. Participants who had measured serum NfL and t-tau levels and data on all components of the CVH score were included. The statistical analysis was conducted from April 10 to September 26, 2024.

**Exposure:**

The CVH score includes 7 components: a healthy diet; regular exercise; normal body mass index; nonsmoking status; and the absence of dyslipidemia, diabetes, and hypertension. The scores were divided into 3 groups from lowest to highest CVH (0-6 points, 7-9 points, and 10-14 points).

**Main Outcomes and Measures:**

The main outcome was the association of CVH score with serum biomarkers of NfL and t-tau as measured using linear regression and mixed-effects models.

**Results:**

A total of 1018 CHAP participants were included in the analysis (mean [SD] age, 73.1 [6.1] years; 625 female [61.4%]; 610 Black or African American [59.9%] and 408 White [40.1%]). Participants with a high CVH score (ie, 10-14 points) were predominantly White (151 [64.3%]) and had a higher education (mean [SD], 13.6 [3.7] years). Compared with participants with low CVH scores (ie, 0-6 points), those with CVH scores of 10 to 14 points had significantly lower serum levels of NfL (relative difference, −18.9%; β = −0.091; SE, 0.025). A higher CVH score was associated with a slower annual increase in NfL levels as participants aged (relative difference in rate, −1.7%; β = −0.008; SE, 0.004). Cardiovascular health was not associated with serum levels of t-tau.

**Conclusions and Relevance:**

These findings suggest that promoting CVH in older adults may help alleviate the burden of neurodegenerative diseases, particularly among Black adults, who are known to experience a higher prevalence of cardiovascular disease.

## Key Points

**Question:**

What is the association between cardiovascular health (CVH) and biomarkers of neurodegeneration, including neurofilament light chain and total tau?

**Findings:**

In this cohort study of 1018 older adult participants (aged ≥65 years) in the Chicago Health and Aging Project, those with high CVH scores had significantly lower serum concentrations of neurofilament light chain, but CVH scores were not associated with total tau concentrations.

**Meaning:**

These findings suggest that promoting CVH in older adults may help reduce the burden of neurodegenerative diseases.

## Teaser

This cohort study investigates whether cardiovascular health is associated with longitudinal changes in biomarkers of neurodegeneration among older adults who participated in the Chicago Health and Aging Project.

## Introduction

The American Heart Association developed a 7-item tool, Life’s Simple 7, to promote cardiovascular health (CVH) in the general population.^1^ Life’s Simple 7 comprises lifestyle and vascular risk factors, including not smoking; maintaining a normal body mass index (BMI); engaging in regular physical activity; consuming a healthy diet; and managing dyslipidemia, diabetes, and hypertension. An optimal CVH characterized by a higher Life’s Simple 7 score is associated with a reduced risk of cardiovascular disease (CVD)^2,3^ and stroke.^4,5,6^ In recent years, there has been increasing research into the association among CVH, cognition, and Alzheimer disease as both CVD and dementia share many common risk factors.^7,8,9^ In particular, a recent study showed that an optimal CVH was associated with a slower rate of cognitive decline, lower risk of Alzheimer disease, and less white matter hyperintensity volumes.^10^ Beyond its impact on brain vascular disease indicators, ie, white matter hyperintensity volumes, CVH may also influence neurodegenerative processes and slow their progression, eventually reducing the risk of Alzheimer disease.^11,12^ Therefore, the aim of this study was to investigate whether CVH is associated with biomarkers of neurodegeneration, including serum levels of neurofilament light chain (NfL) and total tau (t-tau) and if so, whether it is associated with longitudinal changes in these biomarkers over 10 years of follow-up and whether it differs between Black and White individuals.

## Methods

### Study Design and Participants

This cohort study used data from the Chicago Health and Aging Project (CHAP), a longitudinal, biracial, population-based study focused on identifying risk factors for cognitive impairment, Alzheimer disease, and other dementias.^13^ The CHAP study was approved by the institutional review board of Rush University Medical Center, and each participant provided written informed consent. This study followed the Strengthening the Reporting of Observational Studies in Epidemiology (STROBE) reporting guideline.

The CHAP enrolled 10 802 Black or African American (henceforth, Black) and White participants aged 65 years or older from the South Side of Chicago. Data collection was conducted every 3 years, starting in 1993 and continuing until 2012. In each cycle, participants were interviewed at home to obtain information on their demographics and medical history, along with the collection of serum samples. In this study, we focused on 5470 participants who consented to provide serum blood samples. Because of budget limitations, we randomly selected 1327 of these individuals’ samples to undergo immunoassays for NfL and t-tau levels.^14^ Detailed information on the sampling process for assessing biomarkers of neurodegeneration has been previously published.^14,15,16^ From the 1327 individuals with biomarker assessments, we excluded those who did not have all the required components for developing the CVH score (described in the next section). The eFigure in Supplement 1 shows the sample selection process for blood biomarker assessments for this investigation.

### CVH Score

The CVH score was derived from the Life’s Simple 7 definitions (diet, BMI, physical activity, smoking status, dyslipidemia, diabetes, and hypertension).^1^ According to the American Heart Association, a healthy diet includes 5 key components: fruits and vegetables, fish, whole grains, reduced sodium, and reduced sugar-sweetened beverages. Dietary information was collected using a validated 144-item food frequency questionnaire.^17^ Body mass index was calculated by dividing the participant’s weight in kilograms by their height in meters squared and categorized into 3 groups: normal weight (<25), overweight (25-30), and obese (≥30). Physical activity was measured using the 1985 US Health Interview Survey and included questions about moderate or vigorous intensity activities, such as walking for exercise, gardening or doing yard work, performing calisthenics or general exercise, cycling, and swimming.^18^ Smoking status was self-reported and categorized as never, current, or former. A trained research staff member measured systolic blood pressure (SBP) and diastolic blood pressure (DBP) during the in-home interviews, and we defined the presence of hypertension based on blood pressure levels and whether individuals were taking medication for hypertension^10^ (CVH score 0 points: SBP of ≥140 mm Hg and DBP of ≥90 mm Hg; CVH score 1 point: treated blood pressure to SBP <120 mm Hg and DBP <80 mm Hg or SBP of 120-139 mm Hg and DBP of 80-89 mm Hg; CVH score 2 points: untreated blood pressure and SBP <120 mm Hg and DBP <80 mm Hg). Diabetes and dyslipidemia status were assessed through self-report or medication use. During the home interviews, research staff asked participants whether a physician had ever diagnosed them with diabetes, sugar in the urine, or high blood glucose. Additionally, the research staff recorded the participants’ medication use. The use of metformin or insulin was indicative of a diabetes diagnosis, while statin medication indicated dyslipidemia. For the dyslipidemia and diabetes components, we adopted definitions based on data available in CHAP, as described previously.^10^

Each participant was assigned a score of 0, 1, or 2 as determined by their adherence to the 7 cardiovascular health factors. These scores for each factor were then added to generate a total score representing their overall CVH. The CVH score ranged from 0 to 14 points, with higher scores indicating better CVH.

### Demographics and Other Covariates

Demographic characteristics, including age, sex, and race (Black or White, as CHAP was a biracial study), were assessed using 1990 US Census Bureau data.^19^ Education level was self-reported and calculated as years of regular schooling. The apolipoprotein E (apoE) genotypes were measured by the Broad Institute Center for Genotyping, using the homogeneous Mass Extend assay on the Sequenom MassARRAY platform (Agena Bioscience), based on the single nucleotide polymorphisms rs7412 and rs429358.^20^ Heart disease and stroke were defined by participants’ responses to questions from the Established Populations for the Epidemiologic Study of the Elderly.^21^ Creatinine levels were measured in the blood. Cognitive activity was a composite measure ranging from 1 to 5 that included participation in reading, writing letters, visiting a library, and playing games such as chess or checkers. Depressive symptoms were measured using a modified 10-item version of the Center for Epidemiologic Studies Depression Scale.^22,23^

### Serum Concentrations of NfL and t-Tau

Sera collected by a phlebotomist were placed on dry ice for conservation. Next, the sera were transported to the Rush University Medical Center Biorepository Core and stored in a freezer at −80 °C. Furthermore, in 2019, the frozen samples were sent to Quanterix to assess NfL and t-tau concentrations, and the assay kits used for these measurements included the bead-based HD-X Molecular Immunoassay Platform and the Neurology 4-Plex E Assay (Quanterix). Additional information on the measurement of these serum biomarkers has been published previously.^14^

### Statistical Analysis

The characteristics of the study participants are described using means and SDs for continuous variables and the absolute number and percentage for categorical variables. For nonnormally distributed data, we report the median and IQR.

We used linear regression analysis to investigate the association between CVH and the serum neurodegenerative biomarkers NfL and t-tau. The CVH score was assessed as a continuous variable per 1-point increase in the CVH score (range, 0-14 points) and as a categorical variable. For categorical analyses, participant CVH scores were divided into 3 groups from lowest to highest CVH: 0 to 6 points, 7 to 9 points, and 10 to 14 points. The reference category was participants with CVH scores ranging from 0 to 6 points. This categorization of CVH is based on the distribution of people across categories of CVH and aligns with previous published work on the topic.^10^ We also examined the association of CVH score with NfL and t-tau stratified by sex, race, and *APOE* e4 carrier status. To improve the interpretability of the β-coefficient, we back-transformed the log_10_ β-coefficients into a relative difference, which can be interpreted as a percent change (higher or lower) in biomarker levels for the increase of the CVH score as a continuous or categorical variable.^24^

Linear mixed-effects models were used to study the association of CVH score with annual changes in NfL and t-tau levels across the follow-up period among participants with 2 or more measurements of biomarkers in the blood. Models were adjusted for age, sex, race, education, *APOE* e4 carrier status, cognitive activities, depressive symptoms (Center for Epidemiologic Studies Depression Scale score), CVD (stroke and/or heart disease), and follow-up time from the assessment of CVH to the assessment of serum neurodegenerative biomarkers. In addition, as a sensitivity analysis, we adjusted multivariable models by creatinine levels (among participants with these data) to investigate the role of kidney function on associations between CVH and biomarkers of neurodegeneration.

The analyses were conducted between April 10 and September 26, 2024, using R, version 4.2 (R Foundation). A 2-sided *P* < .05 was considered statistically significant.

## Results

A total of 1018 CHAP participants (mean [SD] age, 73.1 [6.1] years; 625 female [61.4%] and 393 male [38.6%]; 610 Black [59.9%] and 408 White [40.1%]) were included in the analysis (Table 1). Participants with the highest CVH score (10-14 points) were predominantly White (151 [64.3%]) and had higher education (mean [SD], 13.6 [3.7] years). A total of 352 participants (34.6%) were carriers of at least 1 *APOE* e4 allele.

**Table 1.** Demographic and Clinical Characteristics of the Study Population and Stratified by CVH Score^a^

Characteristic	Participant group			
	All	CVH score 0-6 points	CVH score 7-9 points	CVH score 10-14 points
No. of participants	1018	230	553	235
Age, mean (SD), y	73.1 (6.1)	72.0 (5.6)	73.2 (6.2)	73.9 (6.3)
Sex, No. (%)				
Female	625 (61.4)	147 (63.9)	327 (59.1)	151 (64.3)
Male	393 (38.6)	83 (36.1)	226 (40.9)	84 (35.7)
Race, No. (%)				
Black or African American	610 (59.9)	184 (80.0)	342 (61.8)	84 (35.7)
White	408 (40.1)	43 (20.0)	211 (38.2)	151 (64.3)
*APOE* e4 carrier, No. (%)	352 (34.6)	84 (36.5)	176 (31.8)	92 (39.1)
Education, mean (SD), y	12.6 (3.6)	11.8 (3.4)	12.5 (3.5)	13.6 (3.7)
Cognitive activities, mean (SD)	3.3 (0.6)	3.1 (0.6)	3.3 (0.6)	3.4 (0.6)
Cardiovascular disease, No. (%)	173 (17.0)	56 (24.3)	86 (15.6)	31 (13.2)
CES-D score, mean (SD)	1.4 (1.8)	1.7 (2.1)	1.4 (1.8)	1.2 (1.7)
BMI, mean (SD)	27.8 (5.5)	30.7 (5.7)	28.0 (5.3)	24.4 (3.4)
Diet score, mean (SD), points^b^	1.0 (0.9)	0.7 (0.7)	0.9 (0.8)	1.4 (0.9)
Physical activity, median (IQR), min/wk	110.0 (5.0-289.4)	0.0 (0.0-98.8)	100.0 (10.0-275.0)	240.0 (151.5-420.0)
Current smoking, No. (%)	112 (11.0)	50 (21.7)	55 (9.9)	7 (3.0)
Systolic blood pressure, mean (SD), mm Hg	137.8 (18.4)	142.6 (18.9)	138.6 (17.9)	131.3 (17.3)
Diastolic blood pressure, mean (SD), mm Hg	77.3 (10.7)	79.1 (12.4)	77.6 (10.0)	75.0 (10.2)
Medication use, No. (%)				
Antihypertensive	564 (55.4)	169 (73.5)	312 (56.4)	83 (35.3)
Antidiabetic	137 (13.5)	94 (40.9)	43 (7.8)	0 (0.0)
Statin	105 (10.3)	63 (27.4)	38 (6.9)	4 (1.7)
Neurodegenerative biomarker, median (IQR)				
Neurofilament light chain, pg/mL	25.4 (18.6-36.4)	26.5 (19.5-37.8)	25.4 (18.1-36.4)	24.6 (19.3-32.8)
Glial fibrillary acidic protein, ng/mL	229.2 (162.0-328.9)	212.2 (156.2-321.4)	230.0 (160.0-327.5)	249.5 (175.8-344.8)
Total tau, pg/mL	0.4 (0.2-0.7)	0.4 (0.2-0.7)	0.4 (0.2-0.7)	0.4 (0.2-0.6)
Lag from CVH to biomarker assessments, mean (SD), y	5.5 (4.0)	5.3 (3.9)	5.8 (4.1)	5.3 (3.8)

Abbreviations: BMI, body mass index (calculated as weight in kilograms divided by height in meters squared); CES-D, Center for Epidemiologic Studies Depression Scale; CVH, cardiovascular health.

^a^ The CVH score was calculated based on recommendations of the American Heart Association’s Life’s Simple 7, with lower scores indicating worse CVH.

^b^ Diet score comprised consumption of 5 food groups, including fruits and vegetables, fish, whole grains, sugar-sweetened beverages, and sodium.

Table 2 shows the association of CVH score with serum concentrations of NfL and t-tau. A higher CVH score (ie, better CVH) was associated with a lower serum concentration of NfL. In a multivariable-adjusted model, a 1-point increase in the CVH score was associated with significantly lower serum levels of NfL (relative difference, −3.5%; β = −0.015; SE, 0.004; *P* < .001). Compared with participants with the lowest CVH scores (0-6 points), those with the highest CVH score (10-14 points) had significantly lower serum levels of NfL (relative difference, −18.9%; β = −0.091; SE, 0.025; *P* < .001). There was no association between CVH and serum t-tau concentrations per 1-point increase in CVH score (β = −0.010; SE, 0.007; *P* = .18). Additional adjustment by creatinine levels for 834 participants (81.9%) showed results similar to those of the primary analysis (eTable in Supplement 1).

**Table 2.** Association of CVH Score With Neurofilament Light Chain and Total Tau^a^

Variable	Neurofilament light chain			Total tau		
	β (SE)	Relative difference, %^b^	*P* value	β (SE)	Relative difference, %^b^	*P* value
CVH continuous						
Per 1-point increase	−0.015 (0.004)	−3.5	<.001	−0.010 (0.007)	−2.2	.18
CVH categorical						
0-6 (low)	Reference			Reference		
7-9	−0.049 (0.020)	−10.7	.01	−0.025 (0.033)	−5.7	.44
10-14 (high)	−0.091 (0.025)	−18.9	<.001	−0.029 (0.041)	−6.5	.47

Abbreviation: CVH, cardiovascular health.

^a^ Models were adjusted for age, sex, race, education, *APOE* e4 carrier status, cognitive activities, depression (Center for Epidemiologic Studies Depression Scale score), and cardiovascular disease (stroke and/or heart disease).

^b^ The relative difference was calculated by back-transforming the log_10_ β-coefficients. A negative value was interpreted as percentage lower and a positive value as percentage higher.

Table 3 shows the association of CVH score with serum concentrations of NfL and t-tau stratified by sex, race, and *APOE* e4 carrier status. We also studied the association of CVH score with NfL and t-tau in participants without CVD at baseline, given that Life’s Simple 7 is mainly promoted among people without CVD. In a multivariable-adjusted model, a 1-point increase in the CVH score was associated with lower serum levels of NfL in both women (β = −0.015; SE, 0.006; *P* = .007) and men (β = −0.017; SE, 0.007; *P* = .01). The association between CVH and NfL did not change by race. A 1-point increase in CVH score was associated with lower serum NfL levels in both Black (relative difference, −3.5%; β = −0.016; SE, 0.005; *P* = .005) and White (relative difference, −3.4%; β = −0.015; SE, 0.007; *P* = .03) participants. A statistically significant association was found between increasing CVH scores and decreasing NfL levels among *APOE* e4 carriers (β = −0.022; SE, 0.007; *P* = .002) but not among *APOE* e4 noncarriers (β = −0.010; SE, 0.005; *P* = .052). With regard to prevalent CVD at baseline, a 1-point increase in CVH score was not associated with lower NfL levels among participants with CVD at baseline (relative difference, −4.2%; β = −0.019; SE, 0.010; *P* = .054) but was significantly lower for those without CVD at baseline (relative difference, −3.1%; β = −0.014; SE, .005; *P* = .005). Baseline CVD did not modify the association between CVH score and NfL, as indicated by the nonsignificant interaction term (*P* = .52).

**Table 3.** Association of CVH Score With Neurofilament Light Chain and Total Tau Stratified by Sex, Race, *APOE* e4 Carrier Status, and Prevalence of CVD^a^

CVH continuous per 1-point increase	Neurofilament light chain			Total tau			
	β (SE)	Relative difference, %^b^	*P* value		β (SE)	Relative difference, %^b^	*P* value
Sex							
Female	−0.015 (0.006)	−3.4	.007		−0.017 (0.010)	−3.8	.08
Male	−0.017 (0.007)	−3.9	.01		0.004 (0.010)	0.8	.72
Race							
Black or African American	−0.016 (0.005)	−3.5	.005		−0.004 (0.009)	−0.8	.69
White	−0.015 (0.007	−3.4	.03		−0.017 (0.011)	−3.9	.13
*APOE* e4 carrier							
No	−0.010 (0.005)	−2.3	.05		−0.016 (0.009)	−3.7	.07
Yes	−0.022 (0.007)	−5.0	.002		0.002 (0.011)	0.4	.88
CVD at baseline							
No	−0.014 (0.005)	−3.1	.005		−0.008 (0.008)	−1.7	.34
Yes	−0.019 (0.010)	−4.2	.05		−0.015 (0.016)	−3.3	.37

Abbreviations: CVD, cardiovascular disease; CVH, cardiovascular health.

^a^ Models were adjusted for age, sex, race, education, *APOE* e4 carrier status, cognitive activities, depression (Center for Epidemiologic Studies Depression Scale score), and CVD (stroke and/or heart disease).

^b^ The relative difference was calculated by back-transforming the log_10_ β-coefficients. A negative value was interpreted as percentage lower and a positive value as percentage higher.

Table 4 shows the association of CVH score with annual changes in serum concentrations of NfL and t-tau. This analysis was focused on 832 participants (81.7%) with follow-up serum NfL and t-tau data. A higher CVH score was associated with a slower annual increase in NfL levels as participants aged. Compared with participants with CVH scores of 0 to 6 points, those with CVH scores of 10 to 14 points had a slower increase in NfL levels per year (relative difference, −1.7%; β = −0.008; SE, 0.004; *P* = .04). The CVH score was not associated with annual changes in t-tau concentrations. The Figure shows the estimated serum levels of NfL and t-tau in participants across CVH scores ranges during the 10-year follow-up. Participants with a CVH score of 0 to 6 points had an annual rate of increase in NfL of 7.1%, and those with a CVH score of 10 to 14 points had an annual rate of increase in NfL of 5.2%.

**Table 4.** Association of CVH Score With Annual Changes in Neurofilament Light Chain and Total Tau

Variable	Neurofilament light chain			Total tau		
	β (SE)	Relative difference, %	*P* value	β (SE)	Relative difference, %	*P* value
CVH continuous						
Per 1-point increase	−0.001 (0.001)	−0.3	.04	0.000 (0.001)	0	>.99
CVH categorical						
0-6 (Low)	Reference	NA	NA	Reference	NA	NA
7-9	−0.005 (0.003)	−1.1	.13	−0.001 (0.005)	−0.2	.85
10-14 (High)	−0.008 (0.004)	−1.7	.04	−0.002 (0.006)	−0.4	.75

Abbreviations: CVH, cardiovascular health; NA, not applicable.

^a^ Models were adjusted for age, sex, race, education, *APOE* e4 carrier status, cognitive activities, depression (Center for Epidemiologic Studies Depression Scale score), cardiovascular disease (stroke and/or heart disease), lag from CVH to biomarker assessment, and interaction of each variable with lag. The interaction of variables with lag allowed us to show the annual changes in neurofilament light chain and total tau serum concentrations.

^b^ The relative difference was calculated by back-transforming the log_10_ β-coefficients. A negative value was interpreted as percentage lower and a positive value as percentage higher.

**Figure.**
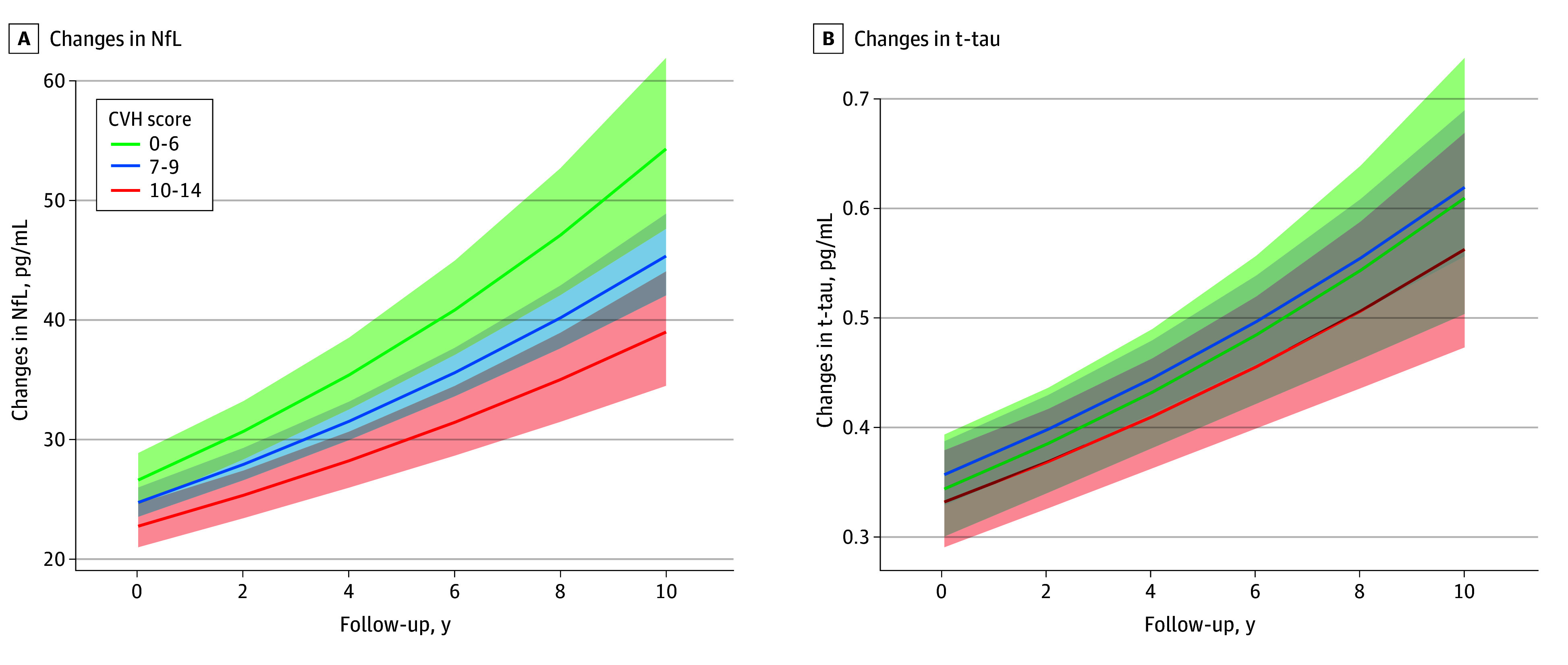
Longitudinal Changes in Serum Levels of Neurofilament Light Chain (NfL) and Total Tau (t-Tau) by Cardiovascular Health (CVH) Group in the Chicago Health and Aging Project A higher CVH score indicates better CVH.

## Discussion

In this prospective cohort study of adults aged 65 years or older, those with better CVH, based on the definition by the American Heart Association’s Life’s Simple 7, had lower serum concentrations of NfL, a biomarker of neurodegeneration. This association was independent of the participant’s age and was similar across men and women, as well as Black and White individuals. Among individuals with a genetic risk of Alzheimer disease, ie, carriers of the *APOE* e4 allele, better CVH was significantly associated with lower serum levels of NfL. The association of CVH and lower serum concentrations of NfL was not modified by the presence of CVD at baseline. Promoting CVH in older adults may help alleviate the burden of neurodegenerative diseases, particularly among Black adults, who are known to experience a higher prevalence of CVD.

The American Heart Association developed the CVH score to promote better CVH in the general population and prevent CVD.^1,25,26,27^ In recent years, there has been increasing research into the association among CVH, cognition, and Alzheimer disease because the heart and brain share similar risk factors, including lifestyle (eg, low physical activity, poor diet, obesity) and vascular (eg, hypertension, diabetes, dyslipidemia).^7,28,29^ Several studies, including ours, have shown that better CVH is associated with a lower risk of incident Alzheimer disease in the general population.^30,31^ For example, in an earlier study using data from the CHAP study, people with better CVH were found to have an approximately 60% lower risk of incident dementia during 18 years of follow-up.^10^ In addition, the study showed that CVH was associated with less white matter hyperintensity volume, suggesting that the mechanistic pathway of CVH for dementia risk may be through vascular pathology.^10^ In the current study, we tested the hypothesis that CVH may also reduce the burden of neurodegeneration in the aging population. We evaluated neurodegeneration by assessing serum concentrations of NfL and t-tau throughout the 10-year study period. We found that better CVH was associated with lower serum concentrations of NfL and a slower increase during the follow-up as the participants aged. These findings suggest that CVH may be associated with the burden of neurodegeneration in the aging population, aligning with another investigation from the Health and Aging Brain Study–Health Disparities (HABS-HD) study.^32^ The HABS-HD study included Mexican American and non-Hispanic White adults and used the Framingham Risk Score to assess CVH. In the HABS-HD study, Mexican American adults with a higher cardiovascular risk score (ie, Framingham Risk Score >20%) had 13.4% increased plasma levels of NfL. Both the HABS-HD and our study underscore the importance of CVH in neurodegenerative diseases and suggest the promotion of CVH as a strategy to reduce the burden of neurodegeneration in the aging population.

Our study showed that while better CVH was associated with lower levels of NfL, there was no significant association of CVH with serum t-tau levels. Both NfL and t-tau are biomarkers of neurodegeneration attributed to many neurodegenerative diseases, including Alzheimer disease.^33,34^ However, increased plasma levels of NfL and t-tau may be attributable to different risk factors and pathways to neurodegenerative diseases. Specifically, NfL is a marker of neuronal damage,^35^ and axonal damage has been, in part, attributed to cerebral injuries, including cerebral vascular pathology.^36^ Cardiovascular health comprises vascular risk factors, such as hypertension and diabetes, known to contribute to vascular damage^37^ and, consequently, compromise blood-brain barrier integrity, thereby increasing neuronal damage and elevating NfL levels. In contrast, t-tau, which primarily reflects tau protein aggregation and neurofibrillary tangles characteristic of Alzheimer disease and other tauopathies,^38^ may not be as directly influenced by CVH status; therefore, we did not find a significant association of CVH with t-tau levels. Regardless, the direction of associations (eg, better CVH and lower concentrations of biomarkers in serum) were similar for both NfL and t-tau in our study. Our findings of CVH, NfL, and t-tau align with the HABS-HD study, which showed that the Framingham Risk Score was more strongly influenced by NfL than t-tau concentrations.^32^

### Strengths and Limitations

A major strength of this study is its prospective longitudinal design in a population setting, with CVH assessed at baseline and biomarkers assessed during follow-up. In addition, we were able to study the changes in biomarkers during the study period as the participants aged. Several components of CVH, such as SBP and DBP, BMI, and medications for hypertension, dyslipidemia, and diabetes, were carefully assessed by trained research personnel.

Our study has several limitations to acknowledge. First, the components of CVH, such as dietary intake, physical activity, and smoking history, relied on self-report, which may introduce misclassification despite the validation of the questionnaires used.^17^ Second, CVH was assessed only at baseline, and we did not explore changes in CVH status during the follow-up period; however, the study design allowed us to evaluate the association with biomarkers prospectively. Third, our findings are primarily derived from older Black and White adults, which may restrict their generalizability to other races and ethnicities. It is important to replicate the findings of this study in other populations to confirm their broader applicability. Fourth, serum biomarkers of neurodegeneration were available only in a subgroup of our study population. In addition to neurodegeneration, NfL is also related to peripheral neuropathies and could, therefore, originate from peripheral sources. The association of CVH with neurodegeneration should not be overstated in this study.^39,40^ Although no association between CVH and t-tau was found, it is important to note that plasma t-tau is considered one of the least reliable biomarkers for Alzheimer disease and may also originate from peripheral sources.^41^ Finally, during the follow-up period, some participants may have developed dementia. While development of incident dementia would not affect our exposure variable (ie, CVH score) measured prior to diagnosis, it may have influenced the sampling process. Participants with severe dementia may have been less likely to consent to providing blood samples. Similarly, participants lost to follow-up due to death; severe illness, particularly those with baseline CVD; or other reasons, such as relocating from the study area, were unable to provide serum samples for neurodegeneration biomarker analysis, potentially influencing the sampling process.

## Conclusions

This cohort study found that individuals with better CVH had lower levels of biomarkers of neurodegeneration, such as NfL, in men, women, Black individuals, and White individuals participating in the CHAP. Promoting CVH in older adults may help alleviate the burden of neurodegenerative diseases, particularly among Black adults, who are know to experience a higher prevalence of CVD.

## Notes

Supplement 1. **eTable.** Association of Cardiovascular Health Score With Neurofilament Light Chain and Total Tau Additionally Adjusted for Kidney Function **eFigure.** Flowchart of CHAP Study Participants for Biomarker Assay Analysis

Supplement 2.
Data Sharing Statement
