# Supplementary material for: Cardiovascular Health and Biomarkers of Neurodegenerative Disease in Older Adults
Source: JAMA Netw Open. 2025 Mar 11;8(3):e250527. doi: 10.1001/jamanetworkopen.2025.0527 (PMC11897837; doi:10.1001/jamanetworkopen.2025.0527)
Supplement: Supplement 1. — eTable. Association of Cardiovascular Health Score With Neurofilament Light Chain and Total Tau Additionally Adjusted for Kidney Function eFigure. Flowchart of CHAP Study Participants for Biomarker Assay Analysis [file jamanetwopen-e250527-s001.pdf]

Supplemental Online Content

Dhana A, DeCarli CS, Dhana K, et al. Cardiovascular health and biomarkers of neurodegenerative disease in older adults. *JAMA Netw Open*. 8(3):e250527. doi:10.1001/jamanetworkopen.2025.0527

eTable. Association of Cardiovascular Health Score With Neurofilament Light Chain and Total Tau Additionally Adjusted for Kidney Function
eFigure. Flowchart of CHAP Study Participants for Biomarker Assay Analysis

This supplemental material has been provided by the authors to give readers additional information about their work.

© 2025 Dhana A et al. *JAMA Network Open*.

eTable. Association of Cardiovascular Health Score With Neurofilament Light Chain and Total Tau Additionally Adjusted for Kidney Function

		Neurofilament light (NfL)		Total tau (t-tau)	
		**Beta ± SE**	**P value**	**Beta ± SE**	**P value**
CVH continuous					
	Per 1-point increase	-0.011 ± 0.004	0.019	-0.003 ± 0.008	0.679
CVH categorical					
	0–6	0.000 (reference)		0.000 (reference)	
	7-9	-0.035 ± 0.021	0.106	-0.011 ± 0.038	0.774
	10-14	-0.063 ± 0.025	0.013	0.003 ± 0.045	0.952

Abbreviations: CVH, cardiovascular health; SE, standard error.
Models were adjusted by age, sex, race, education, APOE e4, cognitive activities, depression (CESD score), creatinine, and cardiovascular disease (stroke and/or heart disease).

© 2025 Dhana A et al. *JAMA Network Open*.

eFigure. Flowchart of CHAP Study Participants for Biomarker Assay Analysis

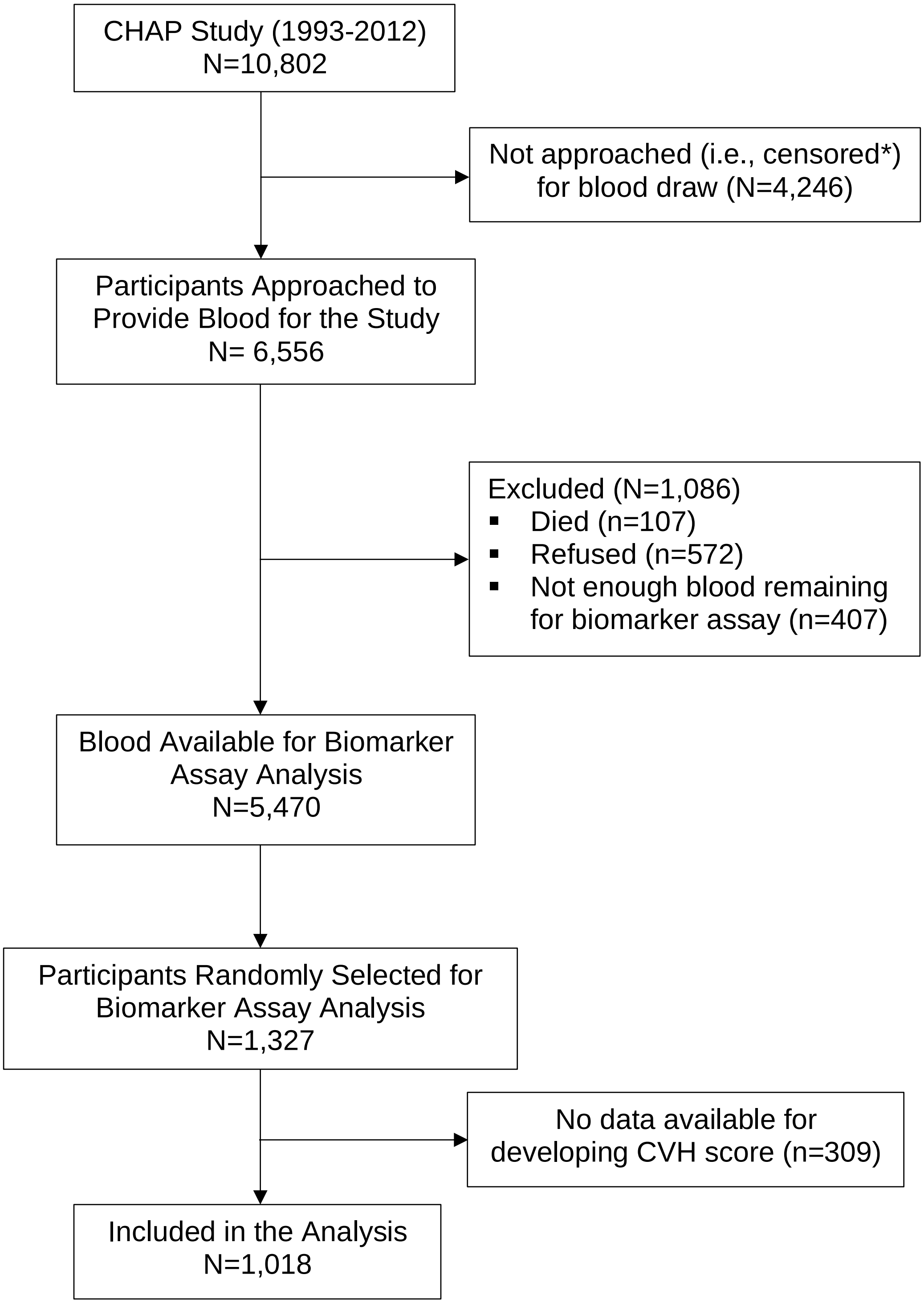

*Censored includes death or lost to follow-up.

© 2025 Dhana A et al. *JAMA Network Open*.
