# Supplementary material for: Cardiovascular Health and Biomarkers of Neurodegenerative Disease in Older Adults
Source: JAMA Netw Open. 2025 Mar 11;8(3):e250527. doi: 10.1001/jamanetworkopen.2025.0527 (PMC11897837; doi:10.1001/jamanetworkopen.2025.0527)
Supplement: Supplement 2. — Data Sharing Statement [file jamanetwopen-e250527-s002.pdf]

Data Sharing Statement

Dhana. Cardiovascular Health and Biomarkers of Neurodegenerative Disease in Older Adults. *JAMA Netw Open*. Published March 11, 2025. doi:10.1001/jamanetworkopen.2025.0527

Data
Data available: Yes
Data types: Deidentified participant data
How to access data: https://www.riha.rush.edu/dataportal/login/registration.htm
When available: With publication

Supporting Documents
Document types: None

Additional Information
Who can access the data: researchers whose proposed use of the data has been approved
Types of analyses: for a specific purpose
Mechanisms of data availability: with a signed data access agreement
